# Genetic variants affecting mitochondrial function provide further insights for kidney disease

**DOI:** 10.1186/s12864-024-10449-1

**Published:** 2024-06-10

**Authors:** Marisa Cañadas-Garre, Blanca Baños-Jaime, Joaquín J. Maqueda, Laura J. Smyth, Ruaidhri Cappa, Ryan Skelly, Claire Hill, Eoin P. Brennan, Ross Doyle, Catherine Godson, Alexander P. Maxwell, Amy Jayne McKnight

**Affiliations:** 1 Molecular Epidemiology and Public Health Research Group, Centre for Public Health,, Queen’s University Belfast, Institute for Clinical Sciences A, Royal Victoria Hospital, Belfast, BT12 6BA UK; 2 Genomic Oncology Area, Centre for Genomics and Oncological Research: Pfizer, GENYO, University of Granada-Andalusian Regional Government, PTS Granada. Avenida de La Ilustración 114, 18016 Granada, Spain; 3 Hematology Department, Hospital Universitario Virgen de Las Nieves, Avenida de Las Fuerzas Armadas 2, 18014 Granada, Spain; 4 Instituto de Investigación Biosanitaria de Granada (Ibs.GRANADA), Avda. de Madrid, 15, 18012 Granada, Spain; 5 Instituto de Investigaciones Químicas (IIQ), Centro de Investigaciones Científicas Isla de La Cartuja (cicCartuja), Consejo Superior de Investigaciones Científicas (CSIC), Universidad de Sevilla, Avda. Américo Vespucio 49, 41092 Seville, Spain; 6 Experimental Oncology Laboratory, IRCCS Rizzoli Orthopaedic Institute, 40136 Bologna, Italy; 7 Department of Experimental, Diagnostic and Specialty Medicine (DIMES), University of Bologna, 40126 Bologna, Italy; 8 UCD Diabetes Complications Research Centre, Conway Institute of Biomolecular and Biomedical Research, University College Dublin, Dublin, D04 V1W8 Ireland; 9 School of Medicine, University College Dublin, Dublin, D04 V1W8 Ireland; 10 Mater Misericordiae University Hospital, Eccles St, Dublin, D07 R2WY Ireland; 11 Regional Nephrology Unit, Belfast City Hospital, Level 11Lisburn Road, Belfast, BT9 7AB UK

**Keywords:** Association, Chronic kidney disease, Diabetic kidney disease, Mitochondrial DNA, Mitochondrial haplogroups, Single nucleotide polymorphisms, UK Biobank

## Abstract

**Background:**

Chronic kidney disease (CKD) is a complex disorder that has become a high prevalence global health problem, with diabetes being its predominant pathophysiologic driver. Autosomal genetic variation only explains some of the predisposition to kidney disease. Variations in the mitochondrial genome (mtDNA) and nuclear-encoded mitochondrial genes (NEMG) are implicated in susceptibility to kidney disease and CKD progression, but they have not been thoroughly explored. Our aim was to investigate the association of variation in both mtDNA and NEMG with CKD (and related traits), with a particular focus on diabetes.

**Methods:**

We used the UK Biobank (UKB) and UK-ROI, an independent collection of individuals with type 1 diabetes mellitus (T1DM) patients.

**Results:**

Fourteen mitochondrial variants were associated with estimated glomerular filtration rate (eGFR) in UKB. Mitochondrial variants and haplogroups U, H and J were associated with eGFR and serum variables. Mitochondrial haplogroup H was associated with all the serum variables regardless of the presence of diabetes. Mitochondrial haplogroup X was associated with end-stage kidney disease (ESKD) in UKB. We confirmed the influence of several known NEMG on kidney disease and function and found novel associations for *SLC39A13*, *CFL1*, *ACP2* or *ATP5G1* with serum variables and kidney damage, and for *SLC4A1*, *NUP210* and *MYH14* with ESKD. The G allele of *TBC1D32*-rs113987180 was associated with higher risk of ESKD in patients with diabetes (OR:9.879; CI_95%_:4.440–21.980; *P* = 2.0E-08). In UK-ROI, *AGXT2*-rs71615838 and *SURF1*-rs183853102 were associated with diabetic nephropathies, and *TFB1M*-rs869120 with eGFR.

**Conclusions:**

We identified novel variants both in mtDNA and NEMG which may explain some of the missing heritability for CKD and kidney phenotypes. We confirmed the role of *MT-ND5* and mitochondrial haplogroup H on renal disease (serum variables), and identified the *MT-ND5*-rs41535848G variant, along with mitochondrial haplogroup X, associated with higher risk of ESKD. Despite most of the associations were independent of diabetes, we also showed potential roles for NEMG in T1DM.

**Supplementary Information:**

The online version contains supplementary material available at 10.1186/s12864-024-10449-1.

## Introduction

Chronic kidney disease (CKD) is increasing in prevalence, especially in older populations, and is a major global health problem [1–4]. CKD is predicted to become the fifth leading cause of death by 2040 [5, 6]. The most severe form of CKD is known as CKD stage 5 or end-stage kidney disease (ESKD), which can be managed by kidney replacement therapy (KRT), such as chronic dialysis and/or kidney transplantation. KRT is expensive, complex, and is often unavailable for persons with ESKD in low- and middle-income countries [7].

CKD is a complex heterogeneous disease whose causality is driven both by genetic and environmental factors. Diabetes and hypertension are major aetiologies contributing to CKD burden [3, 8]. Although CKD heritability can be up to 75% [9–11], molecular markers, identified mainly by meta-analyses of genome-wide association studies (GWAS), do not account for all the inherited susceptibility to CKD [12, 13]. It is therefore plausible that other genetic factors, beyond single nucleotide changes identified from nuclear GWAS, may contribute to CKD [14, 15].

Mitochondria are double-membrane-bound organelles responsible for generating the necessary energy for cellular metabolism [16–18]. Mitochondria contain several copies of their own genome, a circular DNA molecule of ≈16.6 kb in humans including a total of 37 genes: 13 code for the subunits of respiratory complexes I, III, IV and V [19]; 22 code for transfer RNAs (tRNAs) for the 20 standard amino acids, plus an extra gene for leucine and serine [16, 20, 21], and two for ribosomal RNAs (rRNAs) [22]. The replication origin(s) and promoters for mitochondrial DNA (mtDNA) are contained in an additional displacement loop (D-loop). The functions of mitochondria are also regulated by nuclear genes encoding proteins related to mtDNA transcription, replication, cell apoptosis and mitophagy, nucleotide biosynthesis, metabolism, and iron and calcium homeostasis [23, 24].

Mitochondrial dysfunction in kidney tissue can severely impact kidney health and has previously been implicated in CKD development [25–35]. Maintenance of mitochondrial integrity has been highlighted in limiting the progression of acute kidney injury to CKD [36–38]. Lower mtDNA copy number in peripheral blood has been associated with higher risk of diabetes and microalbuminuria, two important risk factors for CKD progression, and with a higher incidence of CKD [39, 40]. Genetic variants in the mitochondrial D-loop have been proposed as predictors of kidney survival in CKD patients, helping to identify CKD patient subgroups at higher risk of poor outcomes [41–44]. Variants in *MT-ND5* have been implicated in adult-onset kidney disease [45]. Genetic variation in mtDNA may be inherited or acquired; human mtDNA is particularly susceptible to acquiring somatic mutations due to its close location to the generation of mutagenic reactive oxygen species through oxidative phosphorylation [46, 47], and the limited nucleic acid repair mechanisms. Higher mutational rates in mtDNA have been reported in tumours, which may correspond to the increased level of reactive oxidative species in renal parenchymal cells in ESKD [48]. Despite the mitochondrial genome being minimally investigated in relation to CKD, some mitochondrial proteins, encoded by nuclear-encoded mitochondrial genes (NEMG), and specific mtDNA variations in *MT-HV2*, *MT-HV3*, *MT-ND5* and *MT-RNR2* have been associated with kidney disease and/or well-established serum clinical biomarkers of CKD, such as serum creatinine (SCr) levels, and estimated glomerular filtration rate (eGFR) [14, 25, 40, 49–51]. Among NEMG, *NAT8*, *CPS1, GATM*, *SLC22A2*, *WDR72* and *AGXT2* are known susceptibility loci for CKD, progression and/or kidney function [52–71].

### Aim

The aim of this study was to investigate the role of genetic variants influencing mitochondrial function on CKD (and related traits). The investigations explore genetic variants in both mtDNA and NEMG. To gain insights into the impact of diabetes on the association of mtDNA and NEMG variants with CKD, we investigated renal phenotypes in a large population cohort (UK Biobank) stratified by diabetes and a collection of individuals with type 1 diabetes mellitus (T1DM) and known kidney function who were explicitly recruited to explore molecular associations with diabetic kidney disease (UK-ROI).

## Methodology

### Study design and populations

**Fig. 1:**
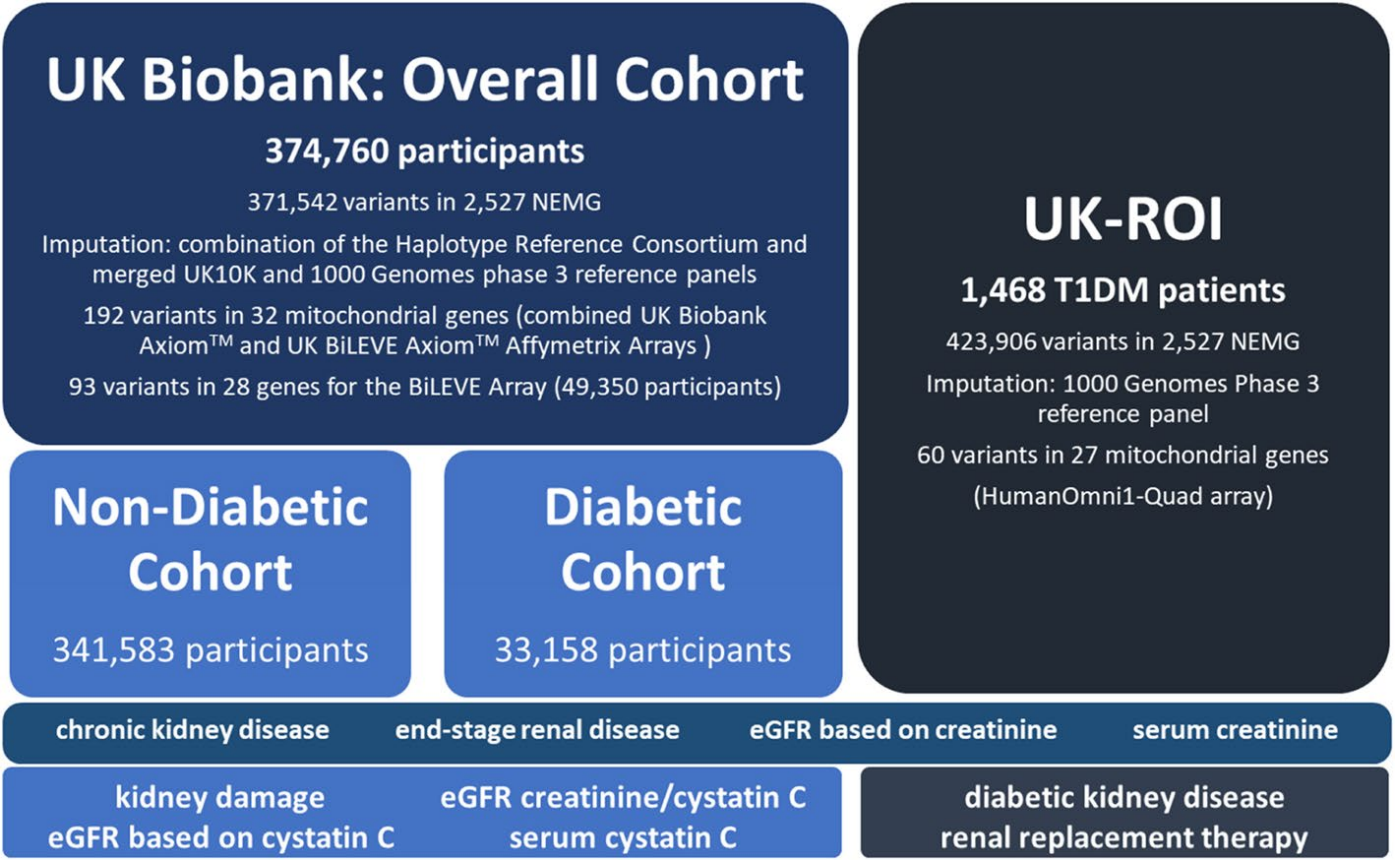
Design of the study, showing final number of participants after quality control, mitochondrial and NEMG variants included, along with the kidney phenotypes studied in each cohort. Abbreviations: eGFR: estimated globular filtration rate. NEMG: nuclear-encoded mitochondrial genes. T1DM: type 1 diabetes mellitus

This cross-sectional study included only participants of European ethnicity with body-mass index (BMI) values within 18.5 and 40 kg/m^2^, corresponding to nutritional status between normal weight and obesity class II, according to the World Health Organisation [72]. Two populations were included in the study (Fig. 1): the UK Biobank (UKB), a population cohort, and the UK-ROI collection of individuals diagnosed with T1DM and known kidney status, as detailed in Supplementary Methods, within the Supplementary Material (Supplementary Methods, Populations). To ensure there was no overlap between these populations, only UK-ROI participants from the Republic of Ireland and Northern Ireland were included.

### Phenotypic variables

Outcome variables included CKD, ESKD, kidney damage (any pathology indicating kidney injury), serum variables, diabetic kidney disease (DKD), and kidney replacement therapy (KRT). Serum variables included serum creatinine (SCr), serum cystatin C (SCysC), eGFR based on SCr (eGFR^Crea^), SCysC (eGFR^CysC^) and SCr/SCysC combined equation (eGFR^CreaCysC^)). Detailed definitions are provided within the ‘Supplementary Methods’ section of the Supplementary Material. Not every variable could be considered for each population (Fig. 1 and Supplementary Table 1).

Analyses were adjusted for age, sex, genotyping batch, smoking habit (defined as ‘yes’ if the patient had ever smoked), hypertension and diabetes (in the analysis of the Overall Cohort for CKD). Data Fields from the UKB used to calculate variables can be found in Supplementary Table 2, along with detailed information on calculations in Supplementary Table 3 and Supplementary Table 4.

### Genotyping and quality control

#### UK Biobank

The Applied Biosystems™ UK Biobank Axiom™ and UK BiLEVE Axiom™ Affymetrix Arrays were used for genotyping by the UK Biobank. Genotypes were imputed by the UKB using a combination of the Haplotype Reference Consortium and merged UK10K and 1000 Genomes phase 3 reference panels [73]. PLINK 1.90 beta and PLINK 2.00 alpha were used to perform quality control (QC) and association analysis [74, 75]. Before QC, the study was comprised of 488,377 participants, 711,188 SNPs in NEMG and 265 mitochondrial directly genotyped variants. Individuals with high missingness rate or call rate lower than 95% were removed. Related individuals (identity by kinship coefficient > 0.0884) and principal component analysis (PCA) outliers, as calculated by the UK Biobank, were also removed [73]. Variants with minor allele frequency (MAF) < 1%, minor allele count (MAC) < 20 or SNP call rate < 95% were removed from the analysis. Autosomal SNPs not fulfilling Hardy–Weinberg equilibrium (HWE) (*p* < 1e-20) or imputation score under 0.3 were also excluded. After QC, 374,760 participants (Overall Cohort), 371,542 variants in 2,527 NEMG, along with 192 variants in 32 mitochondrial genes for the combined arrays and 93 variants in 28 genes for the BiLEVE array remained.

#### UK-ROI

Genotyping of the UK-ROI data collection was performed as described by Sandholm and colleagues [76]. Briefly, DNA samples collected from 1,804 white individuals with T1DM were genotyped with the HumanOmni1-Quad array and imputed to 1000 Genomes Phase 3 reference panel. Before QC, the study was comprised of 1,804 participants, 2,484,108 autosomal variants and 212 mitochondrial DNA directly genotyped variants. Variants with MAF < 1%, MAC < 20 or SNP call rate < 90% were removed from the analysis. Autosomal SNPs not fulfilling HWE (*p* < 1e-20) or imputation score under 0.3 were also excluded. After QC, 1,468 individuals, 423,906 variants in 2,527 NEMG and 60 variants in 23 mitochondrial genes passed filters.

### Mitochondrial haplogroups

Mitochondrial haplogroups were estimated using HaploGrep2 [77], based on PhyloTree17 [78]. Only the major European haplogroups H, V, HV, J, T, U, K, Z, W, X, I and N were considered, grouping the remaining options in the “Other” category.

### Selection of nuclear-encoded mitochondrial genes

A total of 2,527 unique autosomal genes coding for 22,713 transcripts were investigated. The selection process produced 2,448 unique genes returned from database searches with a further 180 genes identified from literature searches for genes influencing mitochondrial function [50]. Briefly, several online databases and literature resources were searched for NEMGs: Mitoproteome [79–82], MitoMiner [83], MitoMap [84], Ensembl [85] and UniProt [86]. Genes extracted from individual sources were reviewed and duplicates were excluded. Gene names were then screened to ensure there was no duplication between the database searches and literature searches. Genes were annotated with their official HUGO Gene Nomenclature Committee (HGNC) gene symbol [87] using Ensembl BioMart release 67 (May 2012) based on the February 2009 Homo sapiens high coverage assembly GRCh37 from the Genome Reference Consortium [85]. Any genes not found in the BioMart [85] search were manually annotated according to their official HGNC gene symbol [87]. The list of genes was then checked again for duplicates based on HGNC symbols, known pseudonyms and gene positions. Only genes found in autosomes were included in the analysis. Any genes on sex chromosomes, non-human genes, or bacterial artificial chromosomes were excluded from the final list of genes encoding proteins required for mitochondrial function.

### eQTL

Tissue data from kidney-cortex used for the analyses described in this manuscript were downloaded from the Portal of The Genotype-Tissue Expression (GTEx) project (GTEx Analysis V8 release; dbGaP accession number phs000424.v8.p2) on 31/01/2022.

### Statistical analysis

 Descriptive analyses were performed using R [88]. Qualitative variables were expressed as percentage (%) of their total. Non-normally distributed variables were expressed as the median and the interquartile range (Q3-Q1). Normality was assessed with Kolmogorov–Smirnov test. 

#### Association analysis

Association analysis for individual variants including sex, age, genotyping batch, smoking habit, hypertension, diabetes (Overall Cohort) and 10 PCAs as covariates was performed with PLINK 2.00 alpha, using the ‘–glm’ flag [74]. For binary phenotypes (CKD, ESKD and kidney damage) –glm fits a logistic or Firth regression model [74]. For quantitative phenotypes, –glm fits the linear model [74]. Quantitative outcome variables were natural logarithmic transformed and analysed using the additional ‘–pheno-quantile-normalize’ flag, to force quantitative phenotypes to a N(0, 1) distribution, preserving only the original rank orders [74]. The *–glm* flag performs a multicollinearity check before each regression, which skips and reports 'NA' results when it fails. Fixed effects meta-analysis was performed for associated variants in the UK-ROI collection showing consistent direction of effect for the same phenotype with UKB using METAL [89]. Between-study heterogeneity was assessed with the I^2^ statistic [90] and random effects meta-analysis was performed if showing a significant heterogeneity (p > 0.05), calculating tau-square and the random effects parameters.

#### Multiple comparisons correction

To correct for multiple testing, a Bonferroni correction for the number of independent variants (estimated using a pruning procedure of our data; r2 < 0.2, window size 50 bp, offset 5 bp) after QC was used [91]. The pruning estimated 47 independent variants for the mitochondrial chromosome for the combined arrays of the UK Biobank (35 when only the BiLEVE array was considered), yielding a threshold of 1E-03, and 57,457 variants for NEMG, yielding a threshold of 9E-07. Our estimation for the mitochondrial chromosome is similar to that calculated by Kraja et al., obtained by permutation analysis, which concluded that 49 variants represented the number of independent genetic effects in the mitochondrial chromosome [92]. For the UK-ROI collection, 60 variants in 23 genes passed QC for the mitochondrial chromosome and 27 were estimated after pruning (0.05/27 = 1.9E-03), with 40,236 variants for NEMG (0.05/40,236 = 1.2E-06).

#### Clumping & annotation

Independent loci were identified using PLINK 1.90 beta clumping procedure (–clump-p1 5e-05 –clump-r2 0.1 –clump-kb 500) [74]. A physical distance threshold for clumping of 1 kb was used for the mitochondrial chromosome. The independent loci were annotated using the variant annotation tool TabAnno [93] complemented with NCBI dbSNP database [94] and SNPnexus [95–99].

#### Mitochondrial haplogroups

Association analysis for mitochondrial haplogroups was performed using logistic regression in R version 3.6.0 (2019–04-26) [88], including as covariates duration of T1DM, sex, hypertension (UK-ROI); age, sex, genotyping batch, diabetes (Overall Cohort), smoking habit and hypertension (UK Biobank). Each haplogroup was analysed separately using all the other haplogroups as reference, after constructing dummy variables taking the values of 0 and 1, with the R package "fastDummies" [100]. Principal components were not used as covariates to account for ancestry because of their potential correlation with haplogroups. The Bonferroni correction was applied to account for multiple comparisons, adjusting the p-value threshold, dividing by the number of haplogroups in each dataset (0.05/number of haplogroups).

## Results

A descriptive analysis of the populations included in the study can be found in Table 1, showing the different strata for the UKB and the diabetic kidney disease (DKD) for the UK-ROI. UKB participants in the subgroup with diabetes were older than those without diabetes and there was a higher percentage of females. In the UK-ROI collection, those with DKD were older and more likely to be males.

**Table 1:** Descriptive analysis of the populations included in the study. For the UK Biobank, a description of the different variables for the overall cohort and the two subgroups after stratification are shown (with and without diabetes)

	**UK Biobank**						**UK -ROI**					
	**Overall cohort**		**Non-diabetic cohort**		**Diabetic cohort**		**All**		**T1DM**		**T1DM + DKD**	
**Variable**	***N***	***n ***(%)	***N***	***n*** (%)	***N***	***n*** (%)	***N***	***n*** (%)	***N***	***n*** (%)	***N***	***n*** (%)
		**mean ± sd**		**mean ± sd**		**mean ± sd**		**mean ± sd**		**mean ± sd**		**mean ± sd**
		**P**_50_ [P_25_-P_75_]		**P**_50_ [P_25_-P_75_]		**P**_50_ [P_25_-P_75_]		**P**_50_ [P_25_-P_75_]		**P**_50_ [P_25_-P_75_]		**P**_50_ [P_25_-P_75_]
**Age** **(years)**	374,741	56.9 ± 8.0	341,583	56.8 ± 8.0	33,158	58.7 ± 7.6	1462	44.3 ± 12.2	750	42.0 ± 12.6	712	46.8 ± 11.3
**Sex** **(male)**	374,741	201,023 (53.6)	341,583	186,110 (54.5)	33,158	14,907 (45.0)	1462	745 (51.0)	750	327 (43.6)	712	418 (58.7)
**Body Mass Index (kg/m**^**2**^**)**	374,741	27.1 ± 4.1	341,583	27.0 ± 4.1	33,158	28.7 ± 4.6	1196	26.3 ± 4.4	648	26.3 ± 4.2	548	26.4 ± 4.7
**Diabetes** **(yes)**	374,741	33,158 (8.9)	341,583	0 (0.0)	33,158	33,158 (100.0)	1462	1462 (100.0)	750	750 (100.0)	712	712 (100.0)
**Ever Smoker** **(yes)**	373,452	169,377 (45.4)	340,457	152,502 (44.8)	32,995	16,869 (51.1)						
**Hypertension** **(yes)**	374,450	295,635 (79.0)	341,308	267,121 (78.3)	33,142	28,498 (86.0)	1378	730 (53.0)	702	54 (7.7)	676	676 (100.0)
**CKD** **(yes)**	373,314	8450 (2.3)	341,583	7022 (2.1)	31,731	1427 (0.0)	1000	507 (50.7)	561	148 (26.4)	439	359 (81.8)
**ESKD** **(yes)**	374,741	89 (0.02)	341,583	69 (0.0)	33,158	20 (0.1)	1000	68 (6.8)	561	0 (0.0)	439	68 (15.5)
**Kidney Damage** **(yes)**	354,218	52,950 (15.0)	322,039	41,729 (13.0)	32,179	11,221 (34.9)						
**eGFR**^**Crea**^ **(mL min-1 per 1.73 m**^**2**^**)**	374,741	92.5 [82.7–99.6]	341,583	92.6 [82.8–99.6]	33,158	92.1 [81.2–99.0]	1000	59.4 [42.0–73.5]	561	68.7 [59.2–82.1]	439	39.2 [22.1–54.0]
**eGFR**^**CreaCysC**^ **(mL min-1 per 1.73 m2)**	374,458	90.6 [81.0–99.6]	341,331	90.8 [81.2–99.7]	33,127	88.9 [78.3–98.5]						
**eGFR**^**CysC**^ **(mL min-1 per 1.73 m**^**2**^**)**	374,458	89.1 [77.4–100.6]	341,331	89.4 [77.8–100.7]	33,127	85.9 [73.2–98.6]						
**SCr** **(mg/dL)**	374,741	0.8 [0.7–0.9]	341,583	0.8 [0.7–0.9]	33,158	0.8 [0.7–0.9]						
**SCysC** **(mg/L)**	374,458	0.9 [0.8–1.0]	341,331	0.9 [0.8–1.0]	33,127	0.9 [0.8–1.0]	1000	1.2 [1.0–1.6]	561	1.1 [1.0–1.2]	439	1.7 [1.3–3.0]
**Duration of T1DM (years)**							1378	30.1 ± 9.7	702	27.4 ± 8.9	676	33.0 ± 9.7
**Kidney Replacement** **Therapy (yes)**							1378	178 (12.9)	702	0 (0.0)	676	178 (26.3)

For the UK-ROI cohort, composed of T1DM patients, a description of the different variables for those with and DKD are shown

*CKD* chronic kidney disease, *DKD* diabetic kidney disease, *eGFR* estimated glomerular filtration rate, *eGFRCrea* eGFR based on serum creatinine, *eGFRCysC* eGFR based on serum cystatin C, *eGFRCreaCysC* eGFR based on the combined serum creatinine and cystatin C equation, *SCr* serum creatinine, *SCysC* serum cystatin C, *ESKD* end-stage kidney disease, *N* total number, *n (%)* frequency (percentage), *sd* standard deviation, *T1DM* type 1 diabetes mellitus. Quantitative variables expressed as mean ± standard deviation or median [interquartile range]. Qualitative variables expressed as frequency and percentages

### Mitochondrial Variants

#### UK Biobank

A higher risk of ESKD was associated with the G allele of the *MT-ND5-*rs41535848 variant in the Non-Diabetic Cohort of the UKB (OR: 4.971; CI_95%_: 1.990–12.415; *P* = 6.0E-04). No other mitochondrial variants were associated with CKD, ESKD or kidney damage in any cohort of the UKB after multiple comparisons correction (*p* < 1E-03).

Fourteen and fifteen mitochondrial variants were associated with eGFR^Crea^ (Supplementary Tables 5 and 10) and eGFR^CreaCysC^ (Supplementary Tables 7 and 12) in the Overall and Non-Diabetic Cohorts, respectively. Ten mitochondrial variants were associated with eGFR^CysC^ in both cohorts (Supplementary Tables 6 and 11).

Fourteen mitochondrial variants in ten genes were consistently associated with eGFR (any equation) both in the Overall and Non-Diabetic Cohorts; additionally, *MT-CO1*-G6734A and *MT-CO3*-rs41482146 were associated with eGFR^Crea^ and *MT-ND4*-rs2853493 with eGFR^CreaCysC^, in the Non-Diabetic Cohort (Supplementary Tables 5–7 and 10–12). None of the mitochondrial variants showed associations in the Diabetic Cohort. Fifteen mitochondrial variants were associated with SCr (Supplementary Tables 8 and 13) and ten with SCysC (Supplementary Tables 9 and 14) in the Overall and Non-Diabetic Cohorts.

#### UK-ROI

No mitochondrial variants were associated with CKD or serum variables after multiple comparisons correction (*p *< 1.9E-03); significant variants from the UKB array rs2854131, rs28359178, rs2853506 and rs2857290 showed consistent directions of effect for eGFR^Crea^ and SCr.

### Mitochondrial haplogroups

#### UK Biobank

**Fig. 2:**
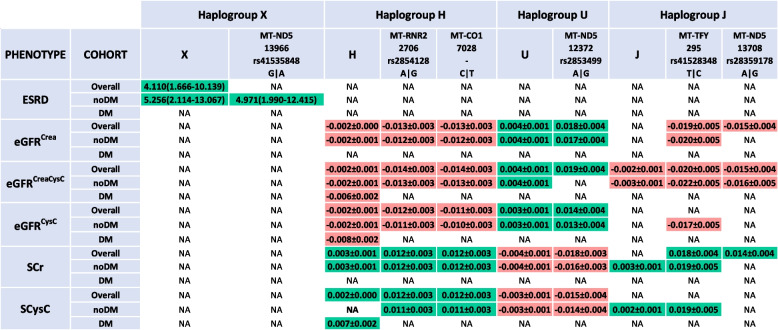
Main results for mitochondrial variants and haplogroups associated with some phenotype in the UK Biobank (Expressed as odds ratio (95% confidence interval) for qualitative variables and betas ± standard error for quantitative variables; Colour code: green increases and red decreases the beta estimate; NA: Not Associated). eGFR^Crea^: eGFR based on serum creatinine. eGFR^CreaCysC^: eGFR based on both serum creatinine and cystatin C. eGFR^CysC^: eGFR based on serum cystatin C. SCr: serum creatinine. SCysC: serum cystatin C. Mitochondrial variants are described as Gene | Base Position (GRCh37) | rs identifier | Effect Allele | Alternative Allele

Six mitochondrial haplogroups were present in the UKB cohort with a frequency over 3% (H, U, J, T, K and I) (Supplementary Table 15). The mitochondrial haplogroups X, H, U, J and I were associated with a number of the phenotypes investigated at the corrected p-value of 0.05/13 = 0.003846 (Supplementary Table 16). Figure 2 shows the main results for mitochondrial variants and haplogroups associated with some phenotype in the UKB.

The mitochondrial haplogroup X, defined by the mutations T6221C, C6371T, A13966G, T14470C, T16189C and C16278T, was associated with higher risk of ESKD in both the Overall and Non-Diabetic cohorts of the UKB, but not in the Diabetic Cohort (Supplementary Table 16), as consistently shown by the association of the A13966G mutation (rs41535848) with ESKD in the Non-Diabetic Cohort (OR: 4.971;CI_95%_: 1.990–12.415; *P* = 6.0E-04).

The mitochondrial haplogroup H, defined by the mutations G2706A (rs2854128) and T7028C, was associated with all the serum variables across all cohorts (Supplementary Table 16), as consistently shown by those two mutations in the Overall and Non-Diabetic cohorts (Supplementary Tables 6–14).

The mitochondrial haplogroup U, defined by the A11467G, A12308G and G12372A mutations, was associated with all the serum variables in both the Overall and Non-Diabetic cohorts (Supplementary Table 16), as seen with the A11467G (rs2853493) for eGFR^CreaCysC^ in the Non-Diabetic cohort (Supplementary Table 12) and G12372A (rs2853499) for all serum variables in the Overall and Non-Diabetic cohorts (Supplementary Tables 6–11;13;14).

The mitochondrial haplogroup J, defined by the C295T, T489C, A10398G, A12612G, G13708A and C16069T mutations, was associated with SCr, SCysC and eGFR^CreaCysC^ in the Non-Diabetic Cohort, but only with the latter in the Overall Cohort (Supplementary Table 16). Consistent associations were found for the individual mutations C295T (rs41528348) and G13708A (rs28359178) (Supplementary Tables 6–14).

The mitochondrial haplogroup I, defined by the mutations T10034C and G16129A, was associated with SCr in both the Overall and Non-Diabetic cohorts (Supplementary Table 16).

The category “Other”, encompassing all the mitochondrial haplogroups not common in Europeans, was associated with SCysC and its derived eGFR equation in both the Overall and Non-Diabetic cohorts (Supplementary Table 16).

#### UK-ROI

The Z mitochondrial haplogroup was not present in the UK-ROI collection, leaving 12 categories to compare (corrected p-value of 0.05/12 = 0.004). The mitochondrial haplogroup J, defined by the C295T, T489C, A10398G, A12612G, G13708A and C16069T mutations, showed a trend to higher risk of kidney replacement therapy (OR: 1.957; CI_95%_: 1.188–3.178; *P* = 0.00727), lower eGFR (BETA: -0.115; CI_95%_: -0.211-(-0.019); *P* = 0.0186) and higher SCr levels (BETA: 0.098; CI_95%_: 0.015–0.181; *P* = 0.0201) although not significant after multiple comparisons correction.

### NEMG

#### UK Biobank

Up to 360 NEMG variants were associated with any serum variable in the UKB dataset (Supplementary Table 17). Among them, only the intronic variant in the NADH:ubiquinone oxidoreductase subunit B10, *NDUFB10*-rs338788C, associated with higher eGFR^Crea^ in the Non-Diabetic Cohort, is a known expression quantitative trait locus (eQTL) in kidney cortex, in particular, increasing the expression of the ribosomal protein L26 (*RPL26*) pseudogene, according to GTEx (Supplementary Table 18). Three SNPs (*SLC39A13*-rs2293576, *LMNA*-rs4641 and *TRMT1*-rs35601737), associated with different kidney phenotypes, are known splicing quantitative trait loci (sQTL) in kidney cortex (Supplementary Table 18). The *SLC39A13*-rs10742802 variant, in strong LD with *SLC39A13*-rs2293576 (R2 = 0.7632) was also associated with Kidney Damage and the serum variables eGFR^Crea^, eGFR^CreaCysC^, SCr and SCysC (Supplementary Table 18).

##### Overall cohort

Five index SNPs were associated with higher risk of CKD with a p-value < 9E-07 in 373,164 participants of the Overall Cohort of the UKB (*NAT8*-rs13538A, *CPS1*-rs1047891A, *SLC22A2*-rs3127573G, *GATM*-rs58764877C, *WDR72*-rs72747347C) (Supplementary Table 19). *NAT8-*rs13538 and *CPS1*-rs1047891 were also associated with all the serum variables in the Overall and Non-Diabetic Cohort, along with *GATM*-rs58764877 for some of the variables. They were also associated with at least one of the serum variables in the Non-Diabetic Cohort. *RAB24*-rs80237806, associated with all the serum variables in the Overall and Non-Diabetic Cohorts, had a p-value for CKD of 2.3E-06.

Two clusters were associated with higher risk of ESKD with a p-value < 9E-07 (*NUP210*-rs144856263T and *SLC4A1*-rs116844389A) for the index SNP in the Overall Cohort of the UKB (Supplementary Table 19).

Twenty-one index SNPs were associated with kidney damage with a p-value < 9E-07 in 352,722 participants of the Overall Cohort of the UKB (Supplementary Table 19), three in common with serum variables.

*CFL1*-rs117624356, *NOS3*-rs3918226 and *SLC39A13*-rs10742802 were associated with all the serum variables in the Overall Cohort; *ACP2*-rs75393320, *ATP5G1*-rs1800632 with SCysC-related variables and *SQOR*-rs629024 with SCr-related variables.

##### Non-diabetic cohort

Four index SNPs were associated with CKD with a p-value < 9E-07 in 340,185 participants of the Non-Diabetic Cohort of the UKB (Supplementary Table 20), three in common with the Overall Cohort (*NAT8*-rs13538, *GATM*-rs58764877 and *WDR72*-rs72747347). *NAT8*-rs13538 was also associated with all the serum variables in the Non-Diabetic Cohort. *GATM*-rs58764877 was associated with some of the serum variables in the Non-Diabetic Cohort.

Two index SNPs (*NUP210*-rs144856263T and *MYH14*-rs148695576T) were associated with higher risk of ESKD with a p-value < 9E-07 in 340,185 participants of the Non-Diabetic Cohort of the UKB (Supplementary Table 20), the first one in common with the Overall Cohort, none in common with serum variables.

Eleven index SNPs were associated with kidney damage with a p-value < 9E-07 in 320,718 participants of the Non-Diabetic Cohort of the UKB (Supplementary Table 20), all in common with the Overall Cohort. Among them, *SLC39A13*-rs10742802 was associated with all serum variables and *NOS3*-rs3918226 and *SQOR*-rs629024 with the SCr-related variables in the Non-Diabetic Cohort.

##### Diabetic cohort

No variants were found to be associated with CKD or kidney damage with a p-value < 9E-07 in 32,979 participants of the Diabetic Cohort of the UK Biobank. Patients with diabetes carrying the G allele of *TBC1D32*-rs113987180 variant (MAF 3%) were at a much higher risk of developing ESKD than patients without diabetes (OR: 9.879; SE: 0.408; *P* = 2.0E-08), whereas the effect in the other UKB cohorts was weaker and non-statistically significant, indicating a potential interaction with the presence of diabetes (Non-Diabetic: OR: 1.226; SE:0.456; *P* = 6.6E-01 and Overall: OR: 2.653; SE: 0.288; *P* = 7.16E-04).

Eleven variants were in common among the different serum variables within the Diabetic Cohort (Supplementary Table 21). In particular, *RBKS*-rs13023003C, *ERBB4*-rs10168303A and *GATM*-15:45672447_GAA_GG (variant included the *GATM*-rs58764877 LD block, associated with CKD in the Overall and Non-Diabetic Cohorts) were associated with most of the variables derived from SCr. These three genotyped variants were associated with higher SCr levels and consequently lower values of eGFR^Crea^. Directions of effects across cohorts were consistent.

#### UK-ROI

Three NEMG variants were associated with at least one phenotype in the UK-ROI collection with a p-value < 1.2e-6 (Supplementary Table 22). The G-allele of *AGXT2*-rs71615838 was associated with lower risk of DKD, *SURF1*-rs183853102A increased the risk of ESKD and *TFB1M*-rs71575026C increased eGFR^Crea^ (Supplementary Table 22).

The *AGXT2*-rs71615838G variant, associated with lower risk of DKD in the UK-ROI collection, showed a different effect in the Diabetic Cohort of the UKB (increased ESKD risk and SCysC levels, decreased eGFR^CysC^ and eGFR^CreaCysC^), but this was not significant after multiple comparisons correction (Table 2).

**Table 2:** Comparison of the values for variants in nuclear-encoded mitochondrial genes associated with any variable in the UK-ROI collection with those in the UKB Cohort (only for associations with *p* < 0.05)

GENE CHR:POS ID Consequence	Population	Phenotype	Cohort	REF	ALT	A1	FREQ (A1)	N	OR or BETA	CI_95%_ or SE	*P*
**AGXT2** **5:35036203** **rs71615838** **Intron**	**UKB**	**ESKD**	Diabetic	G	T	G	0.119	28,344	2.336	4.522—1.207	2.4E-02
		**eGFR**^**CreaCysC**^						32,948	-0.029	0.010	4.8E-03
		**eGFR**^**CysC**^						32,948	-0.036	0.010	6.2E-04
		**SCysC**						32,948	0.037	0.011	7.4E-04
	**UK-ROI**	**DKD**	T1DM	T	G	G	0.050	1377	0.190	0.100—0.360	3.8E-07
**SURF1** **9:136219062** **rs183853102** **Intron**	**UKB**	**CKD**	Overall	A	T	A	0.049	373,164	1.076	1.002—1.155	4.1E-02
		**Kidney Damage**	Non-Diabetic				0.049	320,718	0.963	0.930—0.998	4.1E-02
		**eGFR**^**Crea**^						340,185	0.010	0.005	4.1E-02
		**CKD**	Diabetic				0.048	32,979	1.264	1.070—1.493	6.0E-03
		**eGFR**^**Crea**^						32,979	-0.039	0.016	1.6E-02
		**eGFR**^**CreaCysC**^						32,948	-0.035	0.016	3.0E-02
		**SCr**						32,979	0.042	0.016	7.3E-03
	**UK-ROI**	**ESKD**	T1DM	T	A	A	0.026	999	7.776	3.516—17.199	4.0E-07

*A1* counted allele in regression, *ALT* alternative allele, *BETA* regression coefficient (for A1 allele), *CHR* chromosome, *CI* confidence interval, *DKD* diabetic kidney disease, *eGFR*^Cys^ eGFR based on serum cystatin C, *FREQ* Frequency, *N* number of individuals in the regression, *OR* Odds Ratio (for A1 allele), *P* asymptotic *p*-value (or -log10(p)) for Z/chisq-stat (Qualitative variables) or for T/chisq-stat (Quantitative variables), *POS* base position, *REF* reference allele, *RS* rs identifier, *SCysC* serum cystatin C, *SE* standard error, *T1DM* type 1 diabetes mellitus, *UKB* UK Biobank

The *SURF1*-rs183853102A variant, associated with higher risk of ESKD in the UK-ROI collection, showed consistent effects in the Diabetic Cohort of the UKB (increased CKD risk and SCr levels, decreased eGFR^Crea^ and eGFR^CreaCysC^), but not significant after multiple comparisons correction (Table 2). Combined OR for random effects meta-analysis for ESKD was 4.656 (SE:3.139; *P* = 0.1378; Supplementary Table 23).

The *TFB1M*-rs71575026C variant, associated with higher eGFR^Crea^ in the UK-ROI collection, showed the opposite effect in the UKB cohorts (non-statistically significant).

## Discussion

**Fig. 3:**
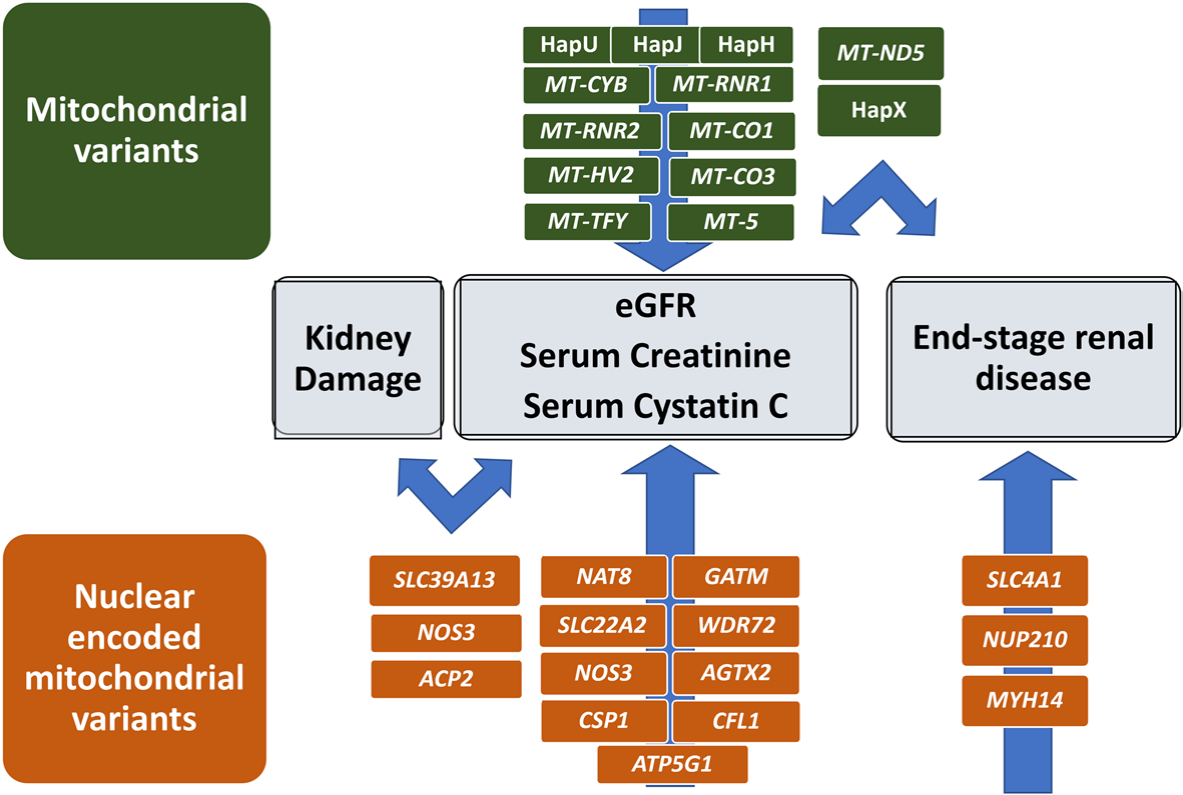
Genes with the associations most consistently shown across cohorts and phenotypes

CKD is a complex heterogeneous disease with a strong genetic component [9–11]. However, CKD heritability is not fully accounted for by current GWAS data [12, 13], indicating that additional genetic factors may be responsible for CKD susceptibility [14, 15]. Mitochondria are crucial to kidney health [25–40], but genetic variation in mtDNA [41–45] and in NEMG [52–70] has not been explored fully [14]. This paper assessed NEMG and mtDNA variants for their association with CKD and kidney phenotypes in individuals with and without diabetes, the most important global risk factor for CKD. Main results are depicted in Fig. 3.

### Mitochondrial variants

*MT-ND5* codes for the mitochondrially encoded NADH:Ubiquinone Oxidoreductase Core Subunit 5, essential for the catalytic activity and assembly of complex I, which catalyses electron transfer from NADH through the respiratory chain, using ubiquinone as an electron acceptor [101]. This gene has been associated with eye diseases as Leber Hereditary Optic Neuropathy (LHON) [102]. Another variant in this gene, rs267606894A (12770A > G), has also been associated with age-related macular degeneration (AMD) in 17,832 controls and 16,144 advanced AMD cases of European ancestry from the International AMD Genomics Consortium (IAMDGC) dataset [103]. In kidney, two *MT-ND5* pathogenic variants, the m.13513G > A and the m.13514A > G, have been involved in adult-onset kidney disease in three unrelated patients [45]. In our study, four polymorphisms in *MT-ND5* (rs2853499, rs28359172, rs2853503 and rs28359178) were associated with eGFR both in the Overall and Non-Diabetic Cohorts. A recent analysis in the UKB by Yonova-Doing et al. showed 16 mitochondrial variants associated with parameters of renal function (SCr, SCysC and eGFR) [51], 10 of them confirmed by our results (*MT-HV2*-rs869183622, *MT-RNR2*-rs2854128, *MT-RNR2*-rs3928306, *MT-RNR2*-rs2854131, *MT-CO1*-7028C, *MT-ND5*-rs2853499, *MT-ND5*-rs2853503, *MT-CYB*-rs2853506, *MT-5*-rs2857290 and *MT-ND4*-rs2853493). *Interestingly, our analysis, not only confirms the association of rs2853499 and rs2853503 variants in MT-ND5 with serum variables, but also newly identifies the MT-ND5-rs41535848G variant, along with the mitochondrial haplogroup X, associated with higher risk of ESKD* in the Overall/Non-Diabetic Cohorts, emphasising the role of this gene in kidney disease. Two variants in *MT-ND5*, G12372A (rs2853499) and G13708A (rs28359178) were associated with practically all serum variables in the Overall and Non-Diabetic cohorts, along with mitochondrial haplogroup U, in line with the study by Yonova-Doing et al. [51]. Another defining mutation for haplogroup U, *MT-ND4*-G11467A (rs2853493), was associated with eGFR^CreaCysC^ in the Non-Diabetic Cohort. *MT-ND4* codes the core subunit 4 of the mitochondrial membrane respiratory chain NADH dehydrogenase (Complex I), which catalyses electron transfer from NADH through the respiratory chain, using ubiquinone as an electron acceptor, essential for the catalytic activity and assembly of complex I [101, 104, 105].

In our study, mitochondrial haplogroup H, along with its defining mutations *MT-RNR2*-G2706A (rs2854128) and *MT-CO1*-T7028C, were associated with all the serum variables across all cohorts, confirming the results by Yonova-Doing et al. for eGFR, SCr and SCysC [51]. The study by Yonova-Doing et al. provided a comprehensive atlas of mitochondrial associations, for a set of 877 complex traits, including kidney phenotypes, but mostly related to kidney function, such as SCr, SCysC and related eGFR [51]. Our study, with a focus on kidney diseases, adds new and specific evidence on CKD, DKD and ESKD and those kidney conditions that may cause injury in the kidneys through the ‘kidney damage’ composite variable. Therefore, our analyses provide more precise evidence on the susceptibility of individuals to kidney disease in different stages and also in a more comprehensive context, as we stratified by diabetes to account for the influence of this condition and identify markers of susceptibility in this specific group of patients.

SNPs in the D-loop were previously identified as potential predictors of kidney survival and poor disease outcomes in 119 CKD patients and 159 controls [41–43]. Among the D-loop mitochondrial variants proposed, the *MT-HV2*-rs869183622A-allele increased CKD susceptibility (99.1% vs 0%; *p* < 0.001) [41], while the analysis in the UKB by Yonova-Doing et al. showed association with SCr and eGFR^CreaCysC^ (*p* < 5·10^–5^) [51]. Our analysis showed a consistent effect of *MT-HV2*-rs869183622A increasing SCr and SCysC levels and decreasing their corresponding eGFR equations.

### NEMG variants

Among the NEMG investigated in our study, *NAT8*-rs13538 and *CPS1*-rs1047891 stood out not only as predictors of CKD, but also showing associations with all the serum variables in the Overall and Non-Diabetic cohorts. The influence of *CPS1*-rs1047891A variant on lower SCr levels and consequently higher scores of eGFR equations based on SCr across was consistent among all cohorts. *NAT8* codes for the N-acetyltransferase 8 (NAT8) enzyme, which catalyses the last step of mercapturic acid formation by acetylating cysteine S-conjugates to mercapturic acids [106] and plays an important role in the development and maintenance of normal kidney and liver structure and function [107], where is abundantly and specifically expressed [107], in particular by tubular cells of the renal cortex [108]. *NAT8* (rs13538) is a known susceptibility locus for CKD and kidney function [13, 52, 54–56, 108–110]. The variant rs10206899 (close to *NAT8* and in LD with rs13538, R2 = 0.988) was associated with SCr, eGFR, SCysC and CKD in a GWAS meta-analysis of nine studies encompassing 23,812 European white participants, by Chambers et al. [108]. *NAT8*-rs13538 results in a non-conservative amino acid change (F143S) within the acetyl-coenzyme A binding site, an effect predicted to influence acetylation by NAT8 [108], a key metabolic pathway for the detoxification of nephrotoxic substances [111, 112]. Association with serum metabolites was reported for *NAT8*-rs13538 (N-acetylornithine) and *GATM*-rs2433610 (located in 15:45,686,091, relatively close to *GATM*-15:45672447_GAA_GG) and *CPS1*-rs1047891 (glycine) in 1,260 African Americans from the Atherosclerosis Risk in Communities (ARIC) study [113]. These SNP-metabolite associations had also been seen in Europeans, in participants of the German KORA F4 study (n = 1,768) and the British TwinsUK study (n = 1,052) [114]. The fact that the risk allele associated here with higher serum levels of N-acetylornithine that is also associated with higher risk of CKD has pinpointed a role for ornithine acetylation in the aetiology of CKD [114]. In support of this, the circulating and urinary levels of 14 N-acetylated amino acids were associated with *NAT8*-rs13538 variant in 962 participants of the African American Study of Kidney Disease and Hypertension, 1050 from the Atherosclerosis Risk in Communities study and 680 from the electronic health record-linked biorepository BioMe [115, 116]. Higher circulating levels of five of these N-acetylated amino acids predicted kidney failure in the combined meta-analysis [115]. However, none of the urinary levels of these N-acetylated amino acids were associated with kidney failure in 1624 participants from the German CKD study [115].

*CPS1* codes for the carbamoyl phosphate synthetase I, the rate-limiting enzyme catalysing the first committed step of the hepatic urea cycle by synthesizing carbamoyl phosphate from ammonia, bicarbonate, and 2 molecules of ATP [117]. *CPS1*-rs1047891, previously associated with eGFR^Crea^ [71, 109], was associated with glycine levels among African Americans from the Atherosclerosis Risk in Communities (ARIC) Study (*P* = 4E-12) [113]. Glycine is metabolically related to carbamoyl phosphate, the product of *CPS1* and the entry point of ammonia into the urea cycle. Other SNPs in this gene were also associated with glycine concentrations in 2,820 individuals from two large population-based European cohorts, as KORA F4 (rs2371015; *P* = 3E-09; R2 = 0.276) and TwinsUK (rs4673553: *P* = 2E-23; R2 = 0.461; rs4673558: *P* = 4.3E-11; R2 = 0.256) [114]. In the meta-analysis, *CPS1*-rs2216405 variant (LD with rs1047891: R2 = 0.385) was also associated with serum levels of glycine, along with serum levels of creatine, produced from glycine, probably indicating an altered ammonia metabolism [114]. Another SNP, located in the 3′ untranslated region of the *CPS1* gene, rs715 (in LD with rs1047891: R2 = 0.907), previously associated with CKD [109], also showed an association with glycine levels in 1,004 nondiabetic individuals from the RISC study (*P* = 3.3E-50) [118]. The influence of this SNP on glycine can be seen not only in serum. In urine samples from 3,861 participants of the SHIP-0 cohort and 1,691 subjects of the KORA F4 cohort, *CPS1*-rs715C was associated with higher glycine/threonine ratio (Beta: 0.141; *P* = 8.5E-31; SHIP-0 dataset) [119]. In the same study, *AGXT2*-rs37369T predicted higher urinary levels of 3-aminoisobutyrate in 3,828 individuals of the SHIP-0 dataset (Beta: 1.277; *P* = 7.5E-26) [119], confirming previous results in urine [120–122] and serum [123]. Other studies had also related this variant with increased serum levels of symmetric/asymmetric dimethylarginine [124] and decreased homoarginine [125]. In our study, a different variant in the same gene, *AGXT2*-rs71615838, predicted higher risk of DKD in T1DM patients from the UK-ROI dataset; however, the effect was not seen in the Diabetic Cohort of the UK Biobank.

In our study, *GATM*-rs58764877 was associated with CKD in the Overall and Non-Diabetic Cohorts, and *GATM*-15:45672447_GAA_GG (in LD with rs58764877) with most of the variables derived from SCr in the Diabetic Cohort. In addition to the *GATM* variant discussed above (rs2433610), another variant in *SPATA5L1*, close to *GATM*, was associated with eGFR^Crea^ in 2,388 CKD cases included among the participants of four different population-based cohorts of European-ancestry (Beta: -0.013; SE: 0.002; *P* = 6.2E-14) [54]. *GATM* encodes the enzyme L-arginine:glycine amidinotransferase, involved in creatine biosynthesis. Although SNPs at this locus have been proposed to affect levels of SCr without influencing susceptibility to kidney disease [54], our results show *GATM* variants can predict both SCr levels and CKD.

In our participants, two variants in *SLC22A2* showed an influence on kidney phenotypes. In particular, *SLC22A2*-rs3127573G increased CKD risk in the Overall Cohort, and *SLC22A2*-rs1554261092(delT) lowered SCr levels, with consequently higher eGFR^Crea^. *SLC22A2* has been previously associated with kidney traits. The *SLC22A2*-rs3127573 variant (located in 6:160,681,393) was associated with SCr and eGFR in the GWAS meta-analysis by Chambers et al. [108]. Other variants as have been associated with eGFR^Crea^ and/or CKD [57, 109]. SLC22A2 codes for the solute carrier family 22 member 2, that are polyspecific organic cation transporters expressed in the liver, kidney, intestine, and other organs and are critical for elimination of many endogenous small organic cations as well as a wide array of drugs and environmental toxins [126].

Genes like *SLC22A2* [57, 108, 109, 127, 128], *CPS1* [109], *WDR72* and *WDR37* [57, 109, 127–129] have shown specific association with eGFR^Crea^, therefore have been proposed to be involved in creatinine secretion, rather than kidney function, and consequently may not be representative of CKD. Functional analysis and other measures of eGFR, such as SCysC, may throw some light on this, to elucidate whether variants associated with creatinine biosynthesis or secretion are really relevant to predict CKD [12]. In our patients, polymorphisms in these three genes were associated with kidney traits not only derived from SCr, but also from SCysC, such as eGFR^CreaCysC^, eGFR^CysC^ and SCysC, along with CKD, showing that their relationship may be indicative of kidney disease and function, not only creatinine production or secretion.

In our study, one of the loci predictive of CKD in both the Overall and Non-Diabetic Cohorts was *WDR72*-rs72747347. Variants in *WDR72* (rs491567; R2 = 0.202 with rs72747347) have been previously associated with eGFR^Crea^ (*P* = 2.7E-13) although not with eGFR^CysC^ [109]. In 14,700 Japanese individuals, *WDR72*-rs10518733 (R2 = 0.193 with rs72747347) was associated with SCr levels (Beta: -0.068; SE: 0.012; *P* = 1.7E-08) [58]. The intronic *WDR72*-rs491567A variant (R2 = 0.202 with rs72747347) decreased eGFR^Crea^ levels in up to 3,282 American Indian individuals from the Strong Heart Family Study (Beta: -0.09; SE: 0.03; *P* = 4.5E-04) [59]. *WDR72*-rs17730436 (R2 = 0.193 with rs72747347) was associated with both SCr (Beta: 0.0005; SE: 0.0007; *P* = 1.2E-13) and eGFR^Crea^ (Beta: -0.0057; SE: 0.0009; *P* = 6E-13) and *WDR72*-rs17730281 (R2 = 0.201 with rs72747347 but R2 = 0.915 with rs17730436) with blood urea nitrogen (BUN) (Beta: 0.0051; SE: 0.0008; *P* = 3E-11) in a GWAS meta-analysis including up to 71,149 East Asian subjects to investigate kidney function-related traits [60]. In 490 unrelated Emirati nationals with type 2 diabetes mellitus (T2DM), variants in *WDR72* showed a trend to association with SCr (rs1031755) or eGFR^Crea^ (rs4776168 and rs10518733), but it was not significant after correction for multiple comparisons (R2 < 0.2 with rs72747347) [61]. The fact that multiple associations within *WDR72* have been shown across different ethnicities suggest that this gene may have relevance for kidney function across multiple ancestries. A recent transethnic GWAS meta-analysis, including participants from the UK and Japan Biobanks, to understand the common genetic factors contributing to nephrolithiasis, identified a potential role of the intronic rs578595 variant in *WDR72* in calcium-sensing receptor (CaSR) signalling [130]. *WDR72*, WD repeat-containing protein 72, encodes a protein with eight WD-40 repeats, highly expressed in kidney [58], which plays an important role in enamel mineralization, possibly due to endocytic vesicle trafficking [131, 132] and is thought to play a role in clathrin-mediated endocytosis, a central process to sustained intracellular CaSR signaling [132]. *WDR72* is also causative of hereditary distal renal tubular acidosis (dRTA), a rare genetic disease, in an autosomal recessive manner [133–135]. One of the genes strongly implicated in the pathogenesis of dRTA is *SLC4A1* [133, 136], encoding for the solute carrier family 4 member 1, part of the anion exchanger family and expressed in the erythrocyte plasma membrane, where it functions as a chloride/bicarbonate exchanger involved in carbon dioxide transport from tissues to lungs. In participants from the Overall Cohort, *SLC4A1*-rs116844389A was associated with ESKD, but not with serum variables. Renal complications of dRTA include nephrocalcinosis, nephrolithiasis, medullary cysts, and impaired kidney function, and it is common to develop moderate to severe CKD over time [137, 138]. *WDR72*-rs77593734T (OR:1.102; *P* = 1.4E-11; R2 = 0.251 with rs72747347) and rs690428A (OR:1.078; *P* = 1.5E-5; R2 = 0.279 with rs72747347) variants were recently identified to increase rapid eGFR^Crea^ decline in a GWAS meta-analysis including 42 studies [139]. In the same study, *WDR72*-rs77593734 was also associated with eGFR^SCysC^ (*P* = 1.9E-16) and BUN in UKB participants [139], confirming previous results in BUN for 416,076 participants from the Chronic Kidney Disease Genetics (CKDGen) Consortium (*P* = 8.5E-17) [71]. In Africans, rs12906891 (R2 = 0.209 with rs72747347) and rs11070992 (R2 = 0.210 with rs72747347) variants in *WDR72* have been associated with proliferative diabetic retinopathy (PDR), a sight-threatening complication of diabetes that is associated with longer duration of diabetes and poor glycemic control (*P* = 9.7E-10; OR:1.46 and *P* = 4.2E-08; OR:1.28, respectively) [140]. *WDR72*-rs551225A (R2 = 0.747 with rs72747347) was associated with low urine pH (Beta: -0.03; CI_95%_: -0.03-(-0.02); *P* = 2.6E-15) and increased risk of kidney stones in a set of 150,274 Icelanders (OR:1.09; CI_95%_: 1.06–1.12; *P* = 4.8E-08) [141].

In our study, *ERBB4*-rs10168303A, an intronic variant located between introns 1–2 of human *ERBB4*, was associated with most of the variables derived from SCr in the Diabetic Cohort of the UKB, showing higher SCr levels and consequently lower values of eGFR. The *ERBB4* gene is a member of the type I receptor tyrosine kinase subfamily, encoding a receptor for NDF/heregulin (NRG1) [142]. Recently, Erbb4-IR, a lncRNA located within the intron region between the first and second exons of *ERBB4* on chromosome 1 of the mouse genome, has been found to induce renal fibrosis [143] and to promote diabetic kidney Injury in *db/db* Mice by targeting miR-29b [144]. This is not the first time that a variant in this gene has been associated with kidney disease in patients with diabetes. The top SNP associated with the primary DKD phenotype identified in a combined GWAS meta-analysis of discovery and second stages in DKD individuals (6, 691) was rs7588550 (R2 < 0.1 with rs10168303), an intronic SNP in the *ERBB4* gene, which demonstrated consistent protective effects in the replication samples (OR = 0.66; CI_95%_: 0.56–0.77; *P* = 2.1E-07) [76].

We also investigated potential eQTLs among our findings. The *SLC39A13*-rs10742802 variant was associated with kidney damage and the serum variables eGFR^Crea^, eGFR^CreaCysC^, SCr and SCysC in both the Overall and Non-Diabetic Cohorts. The *SLC39A13*-rs2293576 variant, in strong LD with *SLC39A13*-rs10742802 (R2 = 0.763) is a known sQTL in kidney cortex. *SLC39A13*, a member of the SLC39A family of zinc transporters, responsible for zinc influx [145], is over-expressed in kidney during dietary zinc deficiency in Wistar rats, playing a role in transporting zinc into cells to avoid zinc depletion [146]. Kidneys contribute to zinc homeostasis in the body by reabsorbing the portion of zinc entering the glomerular filtrate in the nephron [147]. In nonpathological conditions, zinc urinary loss is minimised; however, abnormal kidney function and diabetes, among other conditions, result in reduced serum zinc concentrations and increased urinary excretion [148].

Two other SNPs (*LMNA*-rs4641 and *TRMT1*-rs35601737) associated with different kidney phenotypes in our study, were also found to be sQTL in kidney cortex. The LMNA gene encodes the structural protein components of the nuclear lamina, lamin A and lamin C a protein network underlying the inner nuclear membrane that determines nuclear shape and size. Pathogenic variants in *LMNA* are highly pleiotropic and are responsible for many laminopathies [149] but have also been linked to other unrelated phenotypes, such as CKD (*P* = 1.13E-06), even independently of primary cardiomyopathy (*P* = 1.33E-03) in 11,451 unselected individuals from the Penn Medicine Biobank (Pennsylvania, US), suggesting an independent pathophysiological mechanism for renal failure in the context of loss of function in *LMNA* [150]. Germline *LMNA* mutations carried across generations in focal segmental glomerulosclerosis (FSGS) patients also points to a physiological role of *LMNA* in the maintenance of glomerular structure and function [151]. *TRMT1* has been recently validated as part of a 5-gene prognostic signature for kidney renal clear cell carcinoma (KIRC) in The Cancer Genome Atlas (TCGA) and E-MTAB-1980 cohorts [152, 153].

Among the novel potential associations of NEMG with kidney traits found in our study, the *PTPN11*-rs11614544 variant was consistently associated with SCysC levels and consequently eGFR^SCysC^. Interestingly, *MYH14*-rs148695576T and *NUP210*-rs144856263T were markers associated with a higher risk of ESKD. We also had a particular interest in variants that could identify kidney damage. Among the NEMG predictive of kidney damage along with serum phenotypes in our patients (*NOS3*, *ACP2* and *SLC39A13*), some of them have previously been linked to kidney disease. The transancestry meta-analysis of GWAS for eGFR performed Wuttke et al. [71], using nearly a million individuals, identified 147 loci relevant for kidney function based on associations with the alternative kidney function marker BUN, which subsequently were tested as a GRS in clinically diagnosed CKD and CKD-related outcomes in the UKB. None of the novel signals in our study made it to the GRS in the UKB, but interestingly, *SQOR*-rs629024 and *SLC239A13*-rs10742802 were associated with eGFR^SCr^ (*P* = 7.5E-31 and 1. 9E-10, respectively). Furthermore, when these results were later meta-analysed joining data from the UKB (n = 1,201,909) [154], among their results for eGFR^SCys^, *ATP5G1*-rs1800632 and *PTPN11*-rs11614544 showed p-values of 5.6E-11 and 2.3E-23 respectively, whereas *SQOR*-rs629024 and *SLC239A13*-rs10742802 were confirmed for eGFR^SCr^ (*P* = 7.4E-34 and 1.4E-12, respectively), although these associations were not discussed in the article. The meta-analysis also revealed *NOS3*-rs3918226 associated with eGFR^SCr^ (*P* = 5.4E-09) and *SLC239A13*-rs10742802 with eGFR^SCys^ (*P* = 3.4E-15) [154]. For other variants in our study, there was not information available from previous studies, despite some of the GWAS analysis having been performed on UKB data. There are several reasons why the novel associations identified in our study may not have been seen in previous GWAS of kidney traits. Different study designs, outcome measures and/or different ancestries may have generated different results to our study. The candidate-gene design of our study, including a lower number of variants than a GWAS, has allowed us to use a threshold of 9E-07 to identify associations, lower than the widely accepted GWAS-significant threshold of 5E-08. This may have caused these associations to have been deemed non-significant in such studies. For instance, Kintu et al. recently performed a GWAS meta-analysis to identify susceptibility loci associated with eGFRcrea in 80,027 individuals of African-ancestry from the UKB, Million Veteran Program, and CKDGen consortia [155]. Despite the different ancestry, this study identified 8 lead SNPs, 7 previously associated with eGFRCrea in other populations. However, the authors use a threshold for significance of 5E-08, that may have missed potential variants in common with our study. The summary statistics for these analyses are not available, therefore we cannot confirm whether our associations may have p-values close to the threshold in this case.

It is remarkable that *CFL1*-rs117624356 was associated with all the serum variables in our Overall Cohort, especially given that cofilin-1, encoded by *CFL1*, has been proposed to be integral to the development of proteinuria, being necessary for modulating actin dynamics in podocytes [156, 157]. Podocyte alterations in actin architecture may initiate or aid the progression of glomerular diseases [158]. Cofilin1 has also been involved in hypertensive nephropathy by modulating the nuclear translocation of NF-κB and the expression of its downstream inflammatory factors in renal tubular epithelial cells [159]. Up-regulation of cofilin-1 in HK-2 cells treated with calcium oxalate monohydrate, the major crystalline composition of most kidney stones, suggests it may play a key role in response to the COM crystals adhesion [160]. Animals with mutations in *CFL1* usually display abnormalities including ureter duplication, renal hypoplasia, and abnormal kidney shape [161].

Two variants in *NOS3* (rs3918226 and rs891511) were associated with kidney damage in the UKB cohort. *NOS3*-rs3918226 was also associated with some serum traits. *NOS3* encodes the nitric oxide synthase 3, involved in endothelin pathways and the EGF/EGFR signalling pathway, whose inhibition protects against the development of DKD [162]. There is a clear link between *NOS3* gene variation and ESKD [62, 63], CKD [64] and CKD progression [65]. A recent meta-analysis of 13 studies found that two *NOS3* gene polymorphisms (rs1799983, R2 = 0.189 with rs3918226 and the intron 4 VNTR a/b polymorphism) significantly increased ESKD risk in autosomal dominant polycystic kidney disease (ADPKD) patients [62]. The rs1799983 variant has also showed a role in development of CKD in a recent meta-analysis [64]. The *NOS3*-rs2070744 gene polymorphism (R2 = 0.140 with rs3918226) increased risk of ESKD among 100 Egyptian patients compared to 100 healthy controls (*P* < 0.001) [63]. *NOS3*-rs7830 (R2 = 0.494 with rs891511) has been associated with the risk of DKD in T2DM patients of Greek Caucasian origin (OR: 1.598; CI_95%_: 1.152–2.217; 121 DKD/220 T2DM) [66]. SNPs in *NOS3* have been proposed as potential molecular markers to predict the risk of T2DM and DKD in Chinese Han population [67]. In 490 T2DM patients and 485 healthy controls, *NOS3*-rs3918188 was associated to susceptibility to T2DM; the rs1800783 polymorphism (R2 = 0.1404 with rs3918226) predicted DKD, and family history of diabetes was closely associated with rs11771443 (R2 < 0.1 with rs3918226) polymorphism in DKD [67]. The ACP2 protein was included in a prognostic model of DKD including 35 DKD patients with good and 19 with poor prognosis [68]. On the other hand, associations in *ATP5G1*, the ATP Synthase Membrane Subunit C Locus 1 represents one of the novel findings in our study [62].

One of the aims of our study was to explore the impact of diabetes on the association of mtDNA and NEMG variants with kidney phenotypes. Remarkably, our findings have made evident that the risk of kidney outcomes may be exacerbated by the presence of diabetes. That is the case of *TBC1D32*, not previously linked to kidney traits, accentuating a high risk of ESKD and emerging as a potential predictor of worse outcome in patients with diabetes.

Our study also identified three NEMG predictive of DKD and ESKD among T1DM patients, two of them for the first time. In the UK-ROI collection, individuals carrying the *AGXT2*-rs71615838G variant had decreased DKD risk, whereas carrying the *SURF1*-rs183853102A allele was associated with an increased risk of ESKD. Also, individuals with the *TFB1M*-rs869120C allele had lower serum levels of eGFR. *AGXT2* encodes the enzyme alanine:glyoxylate aminotransferase 2, expressed primarily in the kidney, which converts L-homoarginine (hArg) into 6-guanidino-2-oxocaproic acid (GOCA) [163]. Decreased plasma concentrations of hArg have recently become an emerging marker for clinical status and prognosis in kidney disease [164–166]. In 527 patients with different stages of CKD from the CARE FOR HOMe study confirmed this and found that a decreased ratio between hArg and GOCA predicted even more pronounced risks for kidney events [69]. The mitochondrial aminotransferase encoded by *AGXT2* also catalyses the reaction of β-aminoisobutyrate with pyruvate to form 2-methyl-3-oxopropanoate and alanine.

Overall, our study has found many associations, both in mitochondrial and nuclear genes, with potential implications for the identification of patients with higher susceptibility to kidney diseases. In particular for ESKD, we have identified three genes that might help identify people with a higher risk of developing this more aggressive form of kidney disease, both in the overall population (*NUP210* and *SLC4A1*) and in people with diabetes (*SURF1*). Their actual impact on risk stratification in kidney disease needs further investigation. However, given that most of the effect sizes, especially for the mitochondrial genes, were small, their use in isolation may be limited. To improve kidney disease risk prediction may require carefully validated combinations of clinical risk factors and a variety of ‘-omics’ based predictors.

### Limitations

This is a well-powered cross-sectional study exploring an extensive selection of kidney phenotypes in one of the largest populations cohorts available, the UKB cohort. However, it is not exempt of limitations. The UK-ROI collection and the Diabetic Cohort within the UKB are small compared to the overall UKB cohort and may have reduced the power to detect significant associations. Furthermore, most individuals in the UKB Diabetic cohort had type 2 diabetes, whereas the UK-ROI collection is composed exclusively of T1DM individuals, which may partially explain why findings in T1DM from the UK-ROI collection were not replicated in the UKB.

The eGFR was calculated using the 2009 CKD-EPI creatinine equation, which uses coefficients for age, sex, and race in addition to SCr or SCysC, a premise that has recently come under scrutiny and criticism [167, 168], since race adjustment may have overestimated eGFR (≈16% higher) in Black patients, systematically resulting in an unfavourable bias that potentially may have reduced their access to pre-emptive kidney transplantation. As our study only included participants of European ancestry, no correction for race was applied in the eGFR equations or as covariate in the association analyses, therefore the ethnicity issue should not be a concern.

## Conclusions

In summary, our study has brought to light the influence of variants both in mtDNA and NEMG which may explain some of the missing heritability in CKD. The consistent effects on eGFR and serum variables observed for mitochondrial variants and haplogroups, in particular haplogroup H, associated with all serum variables across all cohorts, support a role of variation in mitochondrial genes for kidney disease (Fig. 3). Our results confirm previously identified associations of NEMG (*NAT8*, *GATM*, *SLC22A2*, *WDR72*, *NOS3, AGXT2* and *CPS1*) with kidney disease and kidney function, providing new information that expands these associations to other kidney disease biomarkers. We also provide new evidence for association of variants in *SLC39A13*, *CFL1*, *ACP2*, or *ATP5G1*, with serum phenotypes and kidney injury. We identified several associations with the most severe stage of CKD—ESKD, both in nuclear (*SLC4A1*, *NUP210 MYH14*) and mitochondrial genes (*MT-ND5*, defining variant for haplogroup X).

Our findings also highlight variants in *TBC1D32* and *SURF1* that are associated with a higher risk of ESKD in individuals with diabetes.

Nevertheless, there are still many ‘gaps’ in our knowledge, beyond SNP variations in nuclear and mitochondrial DNA, to fully understand the genetic predisposition to CKD.

### Supplementary Information

Supplementary Material 1.

Supplementary Material 2.

## Data Availability

The primary data used and/or analysed during the current study are available from the UK Biobank. This research was conducted using the UK Biobank Resource under Application Number 14259. Detailed phenotyping information and summative data are presented in this paper.

## Abbreviations

ADPKD: Autosomal dominant polycystic kidney disease
BMI: Body mass index
CKD: Chronic kidney disease
CKD-EPI: Chronic Kidney Disease Epidemiology Collaboration
DBP: Diastolic blood pressure
DKD: Diabetic kidney disease
D-loop: Displacement loop
DM: Diabetes mellitus
DNCRI: Diabetic Nephropathy Collaborative Research Initiative
eGFR: Estimated glomerular filtration rate
eGFR^Crea^: eGFR based on serum creatinine
eGFR^CreaCysC^: eGFR based on the combined serum creatinine and cystatin C equation
eGFR^CysC^: eGFR based on serum cystatin C
eGFR^EPI^: Estimated glomerular filtration rate based on the CKD-EPI equation
eQTL: Expression quantitative trait loci
ESKD: End-stage kidney disease
GTEx: Gene tissue expression
GWAS: Genome-wide association studies
HGNC: HUGO gene nomenclature committee
HWE: Hardy–Weinberg equilibrium
KRT: Kidney replacement therapy
MAC: Minor allele count
MAF: Minor allele frequency
mtDNA: Mitochondrial DNA
NEMG: Nuclear-encoded mitochondrial genes
PCA: Principal component analysis
QC: Quality control
rRNAs: Ribosomal rRNAs
tRNAs: Transfer RNAs
SBP: Systolic blood pressure
SCr: Serum creatinine
SCysC: Serum cystatin C
SNP: Single nucleotide polymorphism
sQTL: Splicing quantitative trait loci
T1DM: Type 1 diabetes mellitus
T2DM: Type 2 diabetes mellitus
UKB: United Kingdom Biobank
UK-ROI: United Kingdom-Republic of Ireland
