# Supplementary material for: Genetic variants affecting mitochondrial function provide further insights for kidney disease
Source: BMC Genomics. 2024 Jun 10;25:576. doi: 10.1186/s12864-024-10449-1 (PMC11163707; doi:10.1186/s12864-024-10449-1)
Supplement: Supplementary file 1 — Supplementary Material 1. [file 12864_2024_10449_MOESM1_ESM.docx]

# Supplementary Material

## Supplementary Methods

### Populations

#### UKB

The UKB (UKB) is a large study in the United Kingdom which collected genetic and phenotypic information of approximately 500,000 individuals aged between 40 and 69 [1]. This resource facilitates the investigation of diseases and their causes to improve their diagnosis and prevention [2]. ﻿Only individuals with genetic ethnic grouping as Caucasian, as calculated by the UKB, were included in this study [1]. The influence of the gene variants on the different phenotypic outcomes were investigated with/without stratification by diabetes. The total (Overall) cohort was therefore subdivided into two sub cohorts according to the presence (DM) or absence of diabetes mellitus (nonDM).

#### UK-ROI Collection

The UK-ROI collection consists of samples derived from the Republic of Ireland (ROI) and the United Kingdom (Warren 3 and Genetics of Kidneys in Diabetes UK, UK GoKinD), as part of the Genetics of Nephropathy an International Effort (GENIE) and the Diabetic Nephropathy Collaborative Research Initiative (DNCRI) [3] (dbGaP Study Accession: phs000389.v1.p1) [4]. This study population included 1,804 white individuals diagnosed with type 1 diabetes mellitus (T1DM) before 31 years of age, with parents and grandparents born in the UK and Ireland. Full details of these have been previously described [3].

This collection consisted of diabetic kidney disease (DKD) cases which were defined as individuals with persistent proteinuria (>500 mg/24 h) developed more than 10 years after diabetes diagnosis, hypertension (>135/85 mmHg and/or treatment with antihypertensive medication), and retinopathy. Participants were classified as ESKD cases if the individual required kidney replacement therapy (KRT) defined as dialysis or transplantation. Controls were those with no evidence of DKD, including those with persistent normal urine albumin excretion rate (AER; 2 out of 3 urine albumin to creatinine ratio [ACR] measurements <20 µg of albumin/mg of creatinine) despite duration of T1DM for at least 15 years, while not taking any antihypertensive medication, and having no history of treatment with angiotensin-converting enzyme (ACE) inhibitors. The ESKD phenotype was used to gain insight into the influence of mitochondrial genetics in individuals who go on to develop ESKD because of DKD. Normoalbuminuric individuals with T1DM were used as controls to investigate both the DKD and ESKD phenotypes.

### Phenotypic Variables

#### eGFR

The eGFR was calculated using the 2009 CKD-EPI creatinine equation, based on SCr, the 2012 CKD-EPI cystatin C equation, based on SCysC and the 2012 CKD-EPI creatinine-cystatin C equation, as reported by the KDIGO guidelines [5].

#### CKD & ESKD

CKD was defined as eGFR < 60 mL min^-1^ per 1.73 m^2^, and ESKD as eGFR < 15 mL min^-1^ per 1.73 m^2^, according to the KDIGO guidelines [5].

#### Kidney damage

Kidney damage was defined by the presence of any pathology, condition or medication indicative of kidney injury, according to the information provided in the variables non-cancer illness (Data field #20002), participants operations (Data field #20004) and medication (Data field #20003).

#### Hypertension

##### UKB

Participants were considered with hypertension when systolic blood pressure (SBP) > 130 mmHg or diastolic blood pressure (DBP) > 80 mmHg; individuals with medication or conditions related to hypertension were also considered. SBP and DBP were calculated by the average of automatic measures (Data fields #4080 and #4079) or manual measures (Data fields #93 and #94) in its absence. Individuals with blood pressure medication (Data field #6153 and #6177) or other related medications (Data field #20003), non-cancer illness (Data field #20002) and participants operations (Data field #20004) were considered to define hypertension. Details can be found in Supplementary Table 4. Participants were considered to have treated hypertension if they had any record of anti-hypertensive drugs falling in the categories: angiotensin-converting enzyme inhibitors (ACEi), angiotensin II receptor blockers (ARBs), diuretics, calcium channel blockers, beta blockers, alpha blockers and combination drugs from these categories, identified in UKB Data field #20003 (Treatment/medication code). Anti-platelet drugs, anticoagulants, anti-angina medications, anti-arrhythmic agents, statins and medicines for erectile dysfunction or prostate enlargement (which have hypotensive actions) were excluded, since they are not being used directly for management for hypertension. Participants were also considered under anti-hypertensive medication if they had the category “Blood pressure medication” in Data Fields #6177 (Medication for cholesterol, blood pressure or diabetes) or #6153 (Medication for cholesterol, blood pressure, diabetes, or take exogenous hormones). Full list of medications used in the analyses is available on request to the corresponding author.

##### UK-ROI Collection

Participants were considered with hypertension when SBP > 135 mmHg or DBP > 85 mmHg and/or treatment with antihypertensive medication.

#### Diabetes

##### UKB

Participants were considered to have diabetes (either type 1 or 2) if their glycated haemoglobin A1c ≥ 48 mmol/mol (Data Field #30750) or had blood glucose ≥ 7 mmol/L after fasting for > 8 hours (Data Fields #30740, Glucose, and #74, Fasting time); individuals whose diabetes was diagnosed by a doctor (Data field #2443) were also included in the analysis. Other medications (Data field #20003), non-cancer illness (Data field #20002) and participants operations (Data field #20004) were also considered to define diabetes. Details can be found in Supplementary Table .

#### Smoking Habit

##### UKB

Participants were classified as ever had smoked vs never (Data field #20116, never vs previous or current).

# References

1. Bycroft C, Freeman C, Petkova D, Band G, Elliott LT, Sharp K, et al. The UK Biobank resource with deep phenotyping and genomic data. Nature. 2018;562:203–9.

2. Sudlow C, Gallacher J, Allen N, Beral V, Burton P, Danesh J, et al. UK biobank: an open access resource for identifying the causes of a wide range of complex diseases of middle and old age. PLoS Med. 2015;12:e1001779.

3. McKnight AJ, Patterson CC, Pettigrew KA, Savage DA, Kilner J, Murphy M, et al. A GREM1 gene variant associates with diabetic nephropathy. J Am Soc Nephrol. 2010;21:773–81.

4. DbGAP. GEnetics of Nephropathy - an International Effort (GENIE) GWAS of Diabetic Nephropathy in the UK GoKinD and All-Ireland Cohorts. dbGaP Study Accession: phs000389.v1.p1. 2011.

5. Willis K, Cheung M, Slifer S. KDIGO 2012 Clinical Practice Guideline for Evaluation & Management of CKD. Kidney Int Suppl. 2013;3.
